# Case report: The diagnostic dilemma of indeterminate biliary strictures: report on two cases with a literature review

**DOI:** 10.3389/fonc.2024.1301937

**Published:** 2024-03-27

**Authors:** Chunyan Meng, Jing Wang, Peipei Zhang, Bo Wang

**Affiliations:** ^1^ Department of Gastroenterology, Tongji Hospital, Tongji Medical College, Huazhong University of Science and Technology, Wuhan, China; ^2^ Health Management Center, Tongji Hospital, Tongji Medical College, Huazhong University of Science and Technology, Wuhan, China; ^3^ Department of Radiology, Tongji Hospital, Tongji Medical College, Huazhong University of Science and Technology, Wuhan, China

**Keywords:** indeterminate biliary strictures, malignant biliary strictures, cholangiocarcinoma, pancreatic ductal adenocarcinoma, ERCP

## Abstract

**Background:**

It is still a challenging problem for clinicians to explore the nature of the indeterminate biliary strictures (IBSs). Approximately 20% of biliary strictures remain undetermined after a thorough preoperative assessment.

**Case presentation:**

Here, we present two cases of indeterminate biliary strictures patients, whose cross- sectional imaging and endoscopic examination were nondiagnostic. The patients underwent exploratory laparotomy finally and were confirmed as malignancy. We also reviewed the recent reports in literatures regarding the evaluation of IBSs.

**Conclusions:**

Given the majority of the biliary strictures are malignancy, preoperative differentiation between benign and malignant is critical for choosing the best therapeutic regimen. Thus, close follow-up, multiple multidisciplinary discussion, and prompt surgical exploration are necessary for some difficult diagnostic cases.

## Introduction

The term biliary stricture refers to the narrowing in any sites of the hepatic bile duct system, which leads to upstream biliary dilation, and the subsequent obstruction of bile flow. While the majority of patients experience no symptoms with abnormal signs indicated occasionally in imagine examinations, some individuals exhibit only slight symptoms, for example pruritus or jaundice, in their early stage (1).

Biliary strictures develop due to a diverse range of diseases. Benign biliary strictures (BBSs) are relatively uncommon and have various origins. A large proportion of BBSs are following surgery, for instance cholecystectomy or liver transplantation. The incidences of these iatrogenic BBSs are varied from 0.2% to 0.7% and 3% to 13%, respectively. BBBs caused by Chronic pancreatitis (CP) occur in about 13%–21% of patients. Other etiologies include autoimmune diseases like primary sclerosing cholangitis (PSC), IgG4-related sclerosing cholangitis, sarcoidosis, and infectious conditions such as AIDS cholangiopathy (2).

Malignant biliary strictures (MBSs) represent approximately 70% of all bile duct stenosis. Cholangiocarcinoma (CCA) and pancreatic ductal adenocarcinoma (PDA) are the two main etiologies. The proximal and middle bile duct were affected mostly by intra- or extrahepatic CCA, while PDA tends to cause distal bile duct strictures. Less frequently encountered sources of malignant strictures encompass carcinoma of ampulla, gallbladder, liver, or duodena, as well as instances of lymphoma and metastasis to regional lymph nodes (3).

PDA is the most common cause of MBSs. Up to 70% of patients exhibit some degree of MBSs at the time of diagnosis (4). These individuals typically manifest symptoms such as jaundice, weight loss, abdominal pain, often accompanied by a detected mass in the head of the pancreas on imaging modalities or endoscopic ultrasonography (EUS). A hallmark radiographic finding in pancreatic head tumors is the simultaneous dilation of both the pancreatic and common bile ducts, a phenomenon known as the “double duct sign.”

The highly aggressive CCA, arising from the bile duct epithelium, constitutes the second most frequent cause of MBSs. CCA can be classified into intrahepatic, perihilar, or distal types, with perihilar cases accounting for 60 to 70% of occurrences (5). Initially, patients with CCA are usually asymptomatic. However, as the disease progresses, they typically present with symptoms like jaundice, fatigue, and pruritus. Notably, CCA is prevalent among individuals with PSC, a chronic liver disease, characterized by progressive fibrosis and biliary structuring. Up to 15% of patients with PSC will develop CCA, with the highest incidence 2-5 years into diagnosis (6). Unfortunately, CCAs are marked by poor prognoses, with an overall five-year survival rate of less than 20% (5).

Accurate diagnosis is crucial to avoid the omission of malignancy, or unnecessary surgery for benign disease. Despite clinical symptoms combined with biochemical examinations and medical imaging are effective for diagnosing bile duct enlargement and cholestasis, their abilities to evaluate the etiology of biliary obstruction are largely relied on the presence of tissue lumps. In cases of obstructive jaundice where cross-sectional imaging reveals a stricture unaccompanied by discernible mass lesions—referred to as indeterminate biliary strictures (IBSs)—various invasive technologies have been devised to assist in the characterization of the bile duct stenosis (7). Tissue sampling by endoscopic retrograde cholangiopancreatography (ERCP) and/or EUS is a mainstay in the evaluation of IBSs. Increasingly, intraductal ultrasonography (IDUS) and peroral cholangioscopy are emerging technologies employed for direct biopsy sampling. Of note, it is estimated that the nature of approximately 20% of biliary strictures remains undetermined after a thorough preoperative assessment (8).

Here, we demonstrated diagnosis and treatment process of two cases of IBSs, both of which were finally confirmed as malignancy after exploratory laparotomy. We also reviewed the available data on currently developed approaches for the evaluation of IBSs.

## Case presentation 1

**Figure 1:**
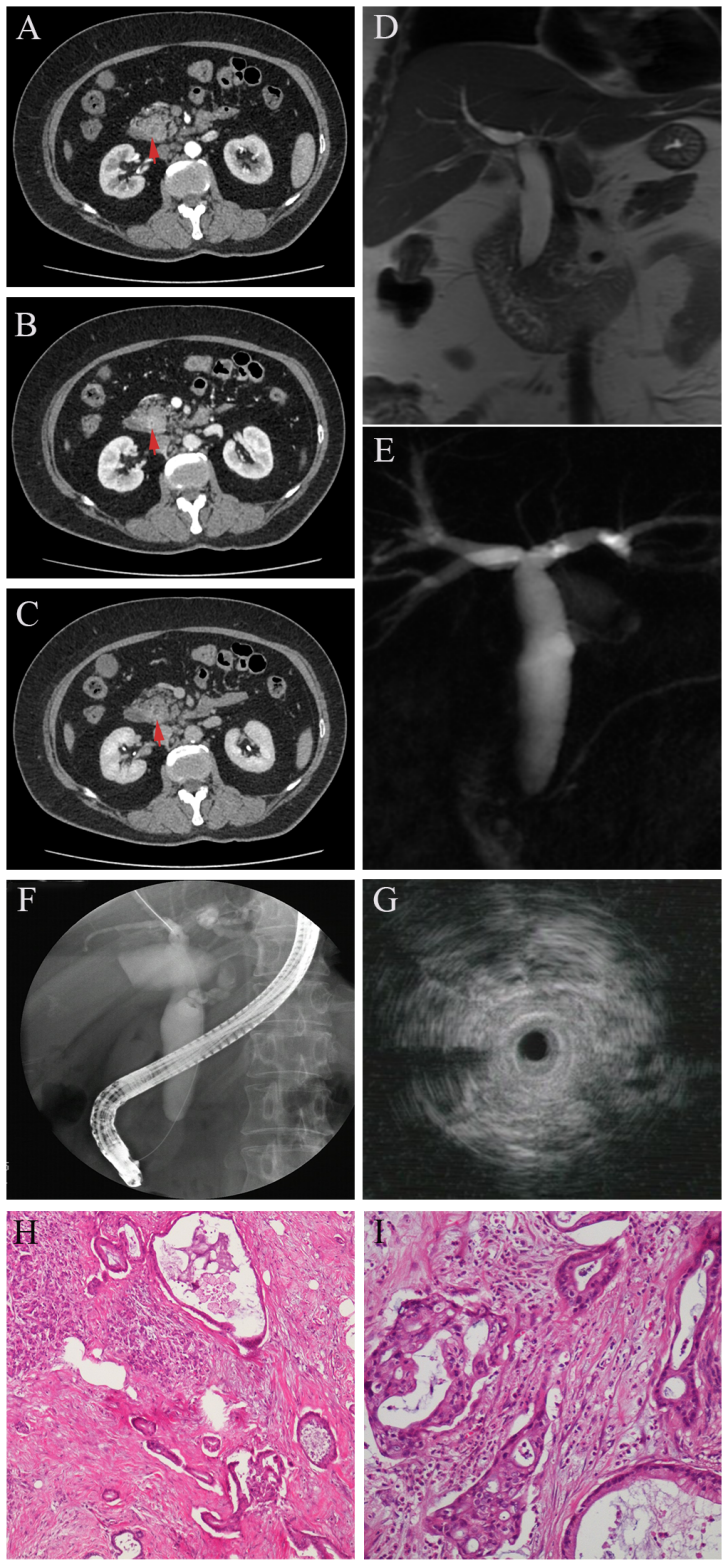
In DCE-CT, the stricture site is not evident in arterial phase (**A**, red arrow), as well as in delayed phase (**C**, red arrow), but enhanced in portal phase (**B**, red arrow). MRCR shows obvious dilation of intra- and extrahepatic bile ducts with distal biliary stricture **(D)** but a non-dilated pancreatic duct **(E)**. Endoscopic Retrograde Cholangiopancreatography (ERCP) revealed a significant concentric stenosis in the lower common bile duct with significant dilatation of the intra- and extrahepatic bile ducts **(F)**. Intraductal ultrasonography (IDUS) showed thickening of the wall in the stenosis site with destruction of normal lumen structure **(G)**. Postoperative pathology revealed distal biliary stricture due to pancreatic adenocarcinoma **(H–I)**.

A 55-year-old woman was referred to our hospital for intermittent right upper quadrant abdominal distention for 2 months and aggravation with jaundice for 1 week. Her medical history included diabetes mellitus treated with metformin and subcutaneous injection with insulin for 5 years. Initial laboratory tests showed compromised liver function (total bilirubin = 5.1 mg/dL, conjugate bilirubin = 4.5 mg/dL) with signs of liver injury (ALT = 199 U/L, AST = 144 U/L, GGT = 741 U/L, ALP = 228 U/L). Of note, her carbohydrate antigen 19–9 (CA19-9) was extremely high (above 1000 U/L), serum globulin was elevated to 40.8 g/L, and serum IgG4 was mildly increased to 2.42 g/L. Other tests, including blood count, coagulation function, erythrocyte sedimentation rate, carcinoembryonic antigen (CEA) and C-reactive protein, were all in normal levels. Abdominal computed tomography (CT) (**Figures 1A–C**, red arrow) and magnetic resonance cholangiopancreatography (MRCP) (**Figures 1D, E**) showed a “rat-tail” stenosis in the intrapancreatic segment of the common bile duct (CBD) and marked dilatation of the biliary tree. The narrow bile duct wall was strengthening in portal phase but normal in arterial and delayed phase in dynamic enhanced CT. Pancreatic duct was normal and no masses were recognizable with radiographic images. Then, ERCP- guided cytobrush and IDUS- guided biopsy were performed in order to further clarify the etiology. ERCP showed the stenosis of the distal bile duct extending over approximately 0.5 cm with upstream dilation of the bile ducts (**Figure 1F**). IDUS demonstrated the destruction of the normal lumen wall at the narrow bile duct without surrounded hypoechoic mass (**Figure 1G**). Cytobrush and intraductal biopsy were taken and the pathological examination results showed only well-differentiated glandular epithelial cells, and no malignant cells. The patient was discharged after liver function and jaundice were improved significantly with effective biliary duct drainage through ERCP. She was re-evaluated 1 month later because of right upper quadrant dull pain and recurrent fever with chills during outpatient follow-up. The patient denied that clinical symptoms were related to diet and anxiety. At this time, most of her laboratory data was improved (total bilirubin = 1.6mg/dL, conjugate bilirubin = 1.1 mg/dL, ALT = 34 U/L, AST = 36 U/L, GGT = 155 U/L, ALP = 205 U/L, globulin = 37.4 g/L, and serum IgG4 was 2.14 g/L), but CA19-9 was still elevated significantly. We performed a new contrast-enhanced CT and MRCP, which revealed the thickening and stricture of distal CBD was similar to the previous results. After multidisciplinary consultation, the patient underwent an laparoscopic exploration with intraoperative cholangioscopy, which demonstrated distal biliary stricture without other obvious detected abnormality. Thus, a resection of the distal bile duct, a Roux-en-Y type hepatic cholangiojejunostomy, and regional lymph node dissections were performed. An examination of the surgical specimens revealed thickening and elongation of the bile duct, with a stenosis length measuring 0.5 cm; however, no discernible mass was observed. However, metastatic tumors were found in the post pancreaticoduodenal lymph nodes. The patient received radical pancreaticoduodenectomy and subsequently chemotherapy with nab-paclitaxel plus gemcitabine after pathological investigations revealed a well differentiated pancreatic ductal adenocarcinoma at the uncinate process of the head of the pancreas (**Figures 1H, I**).

## Case presentation 2

**Figure 2:**
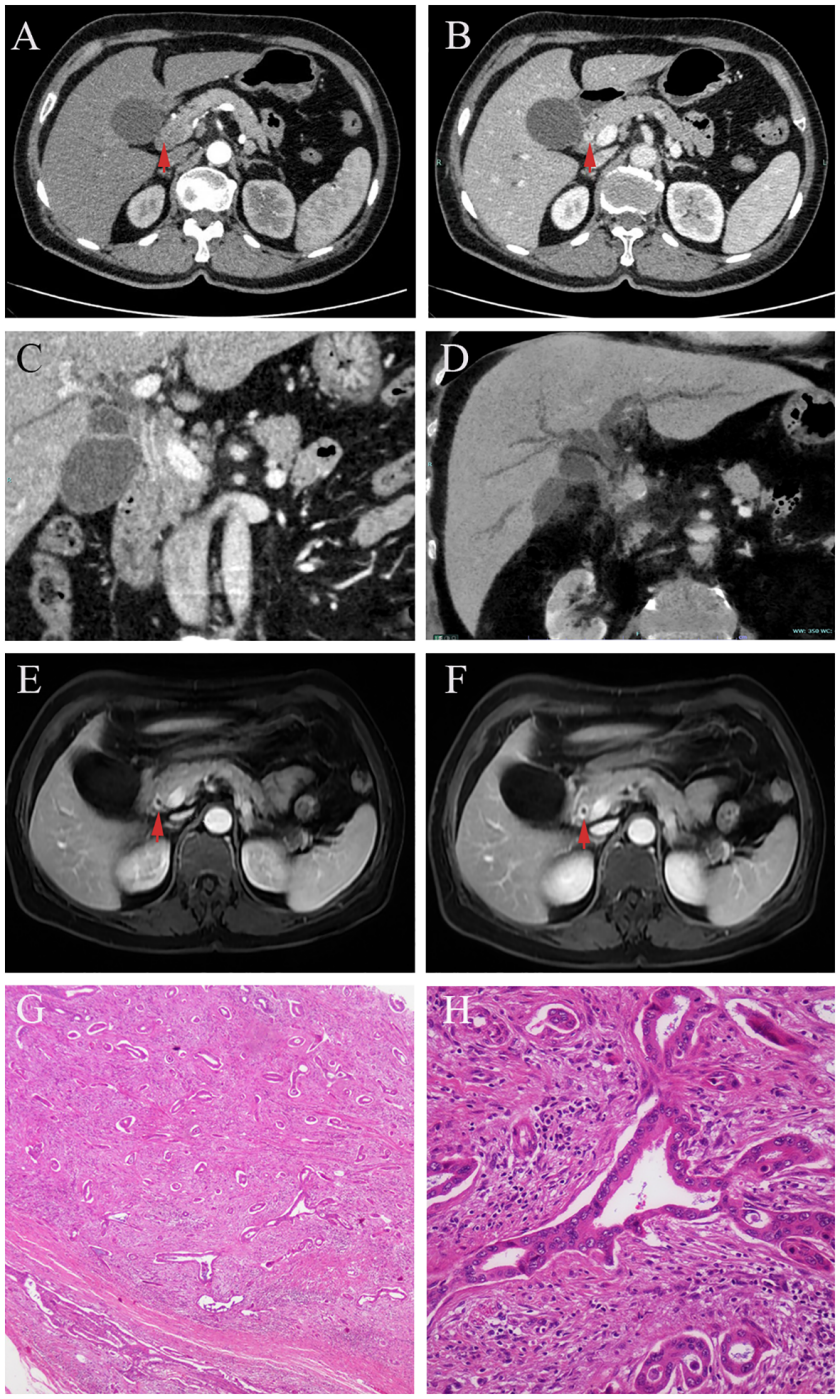
In DCE-CT, the biliary wall is not obviously enhanced in arterial phase (**A**, red arrow), but circular enhancement is seen in portal phase (**B**, red arrow). Multiplanar reconstruction (MPR) in the portal phase of DCE-CT, the wall of the common bile duct is thickened and strengthened at long segments. An abrupt termination of the bile duct with a distal tapering, referred to as a “choledoch cutoff sign” **(C)**. Minimum intensity projection (MinIP) shows the vine-like expansion of the intrahepatic bile duct **(D)**. In DCE-MRI, from the portal phase (**E**, red arrow)to the delayed phase (**F**, red arrow), the enhancement of the bile duct wall becomes evident gradually. Postoperative pathology revealed distal biliary stricture due to cholangiocarcinoma **(G, H)**.

A 65-year-old woman presented to our hospital with a complaint of abdominal pain and jaundice for half a month. She had suffered from icteric hepatitis when she was a child. Physical examination showed marked yellow staining of her skin and sclera without other positive signs. Her laboratory findings revealed abnormal liver function tests (Total bilirubin = 21.5 mg/dL, conjugate bilirubin = 18.7 mg/dL, ALT= 286 U/L, AST = 207 U/L, ALP = 368 U/L, GGT = 1121 U/L). CA19-9 was slightly elevated (64.48 U/mL). CEA and IgG4 (0.207 g/L) were normal. Transabdominal ultrasound showed dilatation of the intra- and extra-hepatic bile ducts, with thickening of the CBD wall and enlargement of the gallbladder. Enhanced CT revealed thickening and enhancement of the common bile duct wall in the portal phase, soft vine dilation of the intrahepatic bile ducts, enlarged gallbladder with non-uniform thickening of the gallbladder wall, and increased perihepatic lymph nodes (**Figures 2A–C**). Multiplanar reconstruction (MPR) of the entire bile duct anatomy showed long segmental stenosis and an abrupt obstruction of the bile duct with gradual tapering of the distal end, known as the “common bile duct cut-off sign” (**Figure 2D**). DCE- magnetic resonance imaging (MRI) showed that the bile duct wall was thickened and gradually strengthened from the portal vein phase to delayed phase (**Figures 2E, F** red arrow). The patient was received antibiotic and other symptomatic treatment. The jaundice abated temporary, but increased progressively with decreased aminotransferase after 1 week. After discussion, the multidisciplinary team considered laparoscopic abdominal exploration. The distal CBD was dissected, and intraoperative fast frozen pathology resulted in the diagnosis of adenocarcinoma. Thus, laparoscopic pancreatico-duodenectomy was performed. The postoperative pathological examination indicated a moderately differentiated adenocarcinoma of the CBD, with the invasion of the entire layer, the gallbladder, and regional lymph nodes (**Figures 2G, H**).

## Discussion

It is frequently a diagnostic dilemma to differentiate the benign from malignant nature of a IBSs patient. Given the prevalence of this condition, it is imperative to conduct a comprehensive assessment for the potential presence of an underlying malignancy in individuals with biliary strictures that cannot definitively be attributed to benign causes. Consequently, a widely adopted principle among clinicians is to approach all biliary strictures with a presumption of malignancy until proven otherwise.

Initial evaluations typically involve non-invasive laboratory and imaging tests, eventually leading to endoscopic procedures with tissue sampling. Among the serum markers utilized for MBSs, CEA has gradually fallen out of favor due to its suboptimal performance. On the other hand, CA19-9 is a prominent marker. At a cutoff value of 37 U/ml, CA19-9 exhibits a sensitivity of 0.77 and a specificity of 0.87 for detecting pancreatic cancer (9). However, on the basis of our descriptive cases and previous literature reviews, the diagnostic value of CA19-9 is not as high in CCA, which may be due to the presence of cholestasis or cholangitis. Some novel biomarkers, such as transthryetin62, matrix metalloproteinase 7, microRNA-21, microRNA-16 and exosomal miRNAs, appear promising in differentiating diagnosis of MBSs (10, 11). Moreover, analyses of proteins, metabolites, and microRNAs using high throughput approach in bile, which so called liquid biopsy, have yielded encouraging results and might be aid to the molecular diagnosis of MBSs (12). The diagnosis value of bile biomarkers in combination with serum CA19-9 seem to be more accurate when facing a challenging patient with IBSs, but must be sufficiently validated in future studies. Serum IgG4 has been measured routinely in IBSs patients in our hospital, since IgG4-related sclerosing cholangitis can present with obstructive jaundice which usually confused with ductal infiltrative CCA. Unfortunately, serum IgG4 level is not elevated in up to 30% of cases of IgG4-related sclerosing cholangitis. Meanwhile, about 15% of CCA, 10% of PSCs, and 5% of the healthy subjects show increased level (13). Therefore, serological indices are far from sufficient to make a diagnosis of MBSs. Imaging test and endoscopy examinations should be performed to further clarify the etiology.

Transabdominal ultrasound demonstrates a notable sensitivity in the detection of intrahepatic branches. However, its ability to clarify MBSs is finite. In general, ultrasonography is very helpful as a screening tool and is advisable to follow it up with higher-resolution imaging examinations (14).

Traditional CT has also limited diagnostic accuracy in characterizing stricture extent. Several modifications in CT scan technologies, such as contrast agents and multidetector CT (MDCT), are helpful for increasing sensitivity of the MBSs. For example, ductal infiltrating CCA typically manifests as strictures without a discrete mass, yet it presents with a hypo-attenuating biliary lesion during the arterial phase and enhancement during the delayed phase in MDCT. Other advantages of MDCT include a comprehensive assessment of adjacent structures of the tumor, and a thorough examination of the entire abdomen to rule out potential metastasis (11). Recently, CT cholangiography imaging obtained through multiplanar reconstruction and minimum intensity projections has emerged as an alternative to MRCP for MBSs assessment, particularly in cases where MRI is contraindicated.

MRI is a promising technique capable of providing an enhanced delineation of fine anatomical structures and small pathological features of the biliary tree. This is very important especially in IBSs, such as MRCP can afford high quality cholangiograms which has similar diagnostic accuracy compared with ERCP, but without a substantial risk of acute pancreatitis. MRCP exhibits notable sensitivity and specificity for distinguishing between malignant and benign forms, ranging from 38% to 90% and from 70% to 85%, respectively. Furthermore, MRCP demonstrates high accuracy (88%-96%) in predicting the extent of bile duct involvement in MBS (15). Morphologically, common imaging features of MBSs include segmental involvement of the biliary tract (>12 mm) and thickening (>1.5 mm) coupled with luminal irregularity, asymmetry, and incremental enhancement (16).

ERCP -guided cytobrush and intraductal biopsy have been considered as the standard approach for the differential diagnosis of IBSs is primarily reliant on the precise delineation of stricture location and extent, as well as tissue acquisition obtained during the procedure. Unfortunately, the approach appears to demonstrate notable specificity while showing limited sensitivity, especially for IBSs, non-bile duct cancer, and short (<30 mm) length of stricture. The poor distinguished value of IgG4-related sclerosing cholangitis from CCA is also the limitation of ERCP. Improvement of sampling techniques, such as combined bile aspiration, cytobrush and biopsy, balloon dilatation before sampling, modified biopsy forceps, wire grasping technique of biopsy, and rapid on-site evaluation (ROSE), have been proved to increase ERCP sensitivity of MBSs. On the whole, all these modifications in the ERCP sampling techniques appear not to reach a satisfactory extent in the diagnostic accuracy in IBSs (17). Nevertheless, technical familiarity, universal availability, perfect specificity, and relief of obstructive jaundice synchronously have contributed to kept these ERCP-based techniques in business over the past decade.

EUS has becoming as an essential means with a good diagnostic yield especially in the evaluation of IBSs in recent years. Compared with cross-sectional imaging, EUS possess higher diagnostic sensitivity for detecting small masses in bile duct and regional lymph nodes, which provided critical clues of diagnostic, prognostic, and therapeutic relevance in cases with MBSs. The accuracy of EUS may be lower in proximal or hilar strictures than distal biliary strictures. Based on the EUS appearance with a pancreatic head mass or a bile duct wall thickness of >3 mm, the sensitivity and specificity of MBSs is 80% and 97%, respectively (18). Meanwhile, sampling acquired through EUS-guided fine needle aspiration (EUS-FNA) could have more benefits than ERCP -guided cytobrush and intraductal biopsy. EUS can also avoid unnecessary ERCP performance and associated complications in IBSs patients without indication of biliary drainage. Noteworthy, the sensitivity of EUS-FNA could achieve 0.77-0.89 in those patients with prior negative ERCP-guided sampling, which suggested the additive value of EUS other than ERCP (19). However, in addition to the advantages mentioned, the potential risks of needle tract seeding and peritoneal dissemination of EUS-FNA have been gradually gathering attention. At present, there is no conclusion as to whether FNA increased peritoneal metastasis or affected overall survival (20). EUS is still recommended as one of the primary modalities for the evaluation of IDBS.

Compared with ERCP- and EUS- guided sampling techniques, IDUS and the single operator cholangioscopes (SOC) have inferior sensitivity by their visual characteristics, both of which need be performed through the working channel of duodenoscope during the index ERCP. IDUS enables a close view of the changes of lumen stenosis. In contrast to EUS, IDUS exhibited superior diagnostic accuracy for proximal obstruction compared to distal bile duct obstruction. The appearance of a destruction of normal three-layered bile duct wall under the high frequency (up to 20 MHz) probe often suggests MBSs. Although IDUS itself cannot perform tissue sampling, it provides useful information for localized intraductal biopsy for IBSs patients after evaluation with EUS and ERCP, which include irregular and eccentric stricture (length >20 mm, thickness>7 mm) and mass invasion into surrounding structures (21).

With respect to SOC, it has been in extensive use in diagnosis for IBSs since it overcame problems of poor reliability and the inconvenient operation of old mother-baby endoscope system. Designed to directly visualize the stricture of bile ducts during diagnostic and therapeutic procedures, SOC is significantly superior in providing powerful insight to increase diagnostic sensitivity and specificity. SOC predicts occult malignancy through various morphological elements of the strictures including tumor vessels, papillary projection, nodular or polypoid mass, and infiltrative lesions (22). A multicenter prospective study published in 2020 reported its specificity of 68%, and sensitivity of 86% based on the visual features of biliary malignancy (23). These results were reinforced by another meta-analysis that reviewed a overall pooled specificity and sensitivity of 95% and 94%, respectively (24). Despite visual impression of SOC has a high sensitivity, histopathology results of biopsy on the target site remain to be the gold standard for diagnosis. Gerges and his colleagues compared SOC-guided biopsy (SB) with ERCP-guided brushing (EB) in the IBS patients and found the distinct preponderance of SB on sensitivity (SB 68.2% *vs* CB 21.4%) and overall accuracy (SB 87.1% *vs* CB 65.5%). Nevertheless, the specificity, positive or negative predictive value and adverse events were of great resemblance (25). In light of the above, it is important to note as well that limitations of SOC continue to be a main concern of the clinicians. As a first priority there has been a lack of interobserver agreement when applying visual findings, which support the need for formally established and validated visual criteria (26). Cost is in the second place, and additionally the associated complications, such as cholangitis and pancreatitis. Potential strategies for optimizing the outcomes of SOC include operator’s experience, timepoint of biliary stent placement, ROSE of touch imprint cytology and new cholangioscopic classification system. Looking into the future, we expect to see enhancement of the accuracy of SOC and the mitigate interobserver variability through the growing integration of artificial intelligence on image interpretation (27).

In recent years, numerous novel technologies have been introduced in the evaluation of IBSs, most of which are based on the successful biliary duct cannulation during ERCP. Confocal laser endomicroscopy (CLE) can offer real-time microscopic insights into biliary structures. The sensitivity and specificity for detecting biliary malignancies was estimated at 0.88, and 0.79, respectively (28). Specimens obtained from ERCP can be used for fluorescence *in situ* hybridization (FISH) or next-generation sequencing (NGS) for analyzing a multitude of genes frequently altered through mutations, amplifications, and/or deletions in malignancies affecting the biliary tract (29).

## Conclusion

Overall, definite diagnosis of IBSs has been difficult so far, even after performed all of the above assessments. Emerging technologies of this nature are not yet widely accessible and necessitate additional validation before they can be incorporated into the assessment of biliary strictures in the future. Given MBSs is still the main cause of IBSs, close follow-up, multiple multidisciplinary discussion, and prompt surgical exploration are necessary for some difficult diagnostic cases.

## Data availability statement

The original contributions presented in the study are included in the article/supplementary material. Further inquiries can be directed to the corresponding author.

## Ethics statement

Written informed consent was obtained from the individual(s) for the publication of any potentially identifiable images or data included in this article.

## Author contributions

BW: Investigation, Supervision, Writing – original draft, Writing – review & editing. CM: Data curation, Investigation, Writing – original draft. JW: Writing – original draft, Writing – review & editing. PZ: Formal analysis, Writing – original draft.
